# Modernizing public health communication competencies in Canada: A survey of the Canadian public health workforce

**DOI:** 10.17269/s41997-024-00890-w

**Published:** 2024-05-16

**Authors:** Devon McAlpine, Melissa MacKay, Lauren E. Grant, Andrew Papadopoulos, Jennifer E. McWhirter

**Affiliations:** Department of Population Medicine, University of Guelph, Guelph, Ontario Canada

**Keywords:** Health communication, Public health, Professional competence, Public health education for professionals, Communication sur la santé, santé publique, compétence professionnelle, formation professionnelle en santé publique

## Abstract

**Objectives:**

Since the publication of the Core Competencies for Public Health in Canada in 2008, the public health and communication landscape has changed dramatically. Digital media and infodemics have shifted how practitioners must communicate and respond to health information. The age of the current competency framework, which is relied on for workforce development, alongside emerging public health challenges, have prompted calls for modernized competency statements. This study aims to (i) measure self-reported communication competence in the public health workforce, (ii) measure agreement with new communication competency statements, (iii) identify variation in agreement between sub-groups of professionals, and (iv) explore current and needed communication training.

**Methods:**

Using a mixed-methods online survey, a sample of 378 participants in various Canadian public health roles and regions were asked to rate their current communication competence and agreement with a modernized, evidence-based draft communication competency framework. The survey was distributed in both official languages through partner organizations and social media. Descriptive statistics were performed to assess agreement and variation was analyzed in relation to public health roles and experience.

**Results:**

While most participants self-reported communication competence, specific areas were rated lower. All 21 proposed competency statements received high agreement with some variation observed between expertise and experience levels. Demand for communication training is high.

**Conclusion:**

Strong agreement with statements indicates support for a modernized communication competency framework among sampled professionals. Research to gather more evidence surrounding the communication demands of the public health workforce and observed variation in strong agreement for the proposed statements is underway.

**Supplementary Information:**

The online version contains supplementary material available at 10.17269/s41997-024-00890-w.

## Résumé

**Objectifs:**

Depuis la publication des Compétences essentielles en santé publique au Canada en 2008, le paysage de la santé publique et des communications a radicalement changé. Les médias numériques et l’infodémie ont fait évoluer les façons dont les praticiennes et les praticiens doivent communiquer les informations sur la santé et y réagir. L’âge du cadre de compétences actuel et son importance pour le développement de la main-d’œuvre, en plus des problèmes de santé publique émergents, suscitent des appels à moderniser les énoncés de compétences. Notre étude vise à : i) mesurer la compétence en communication autodéclarée dans la main-d’œuvre en santé publique, ii) mesurer l’accord avec de nouveaux énoncés de compétences en communication, iii) déterminer si les sous-groupes professionnels diffèrent dans leurs niveaux d’accord et iv) explorer la formation en communication actuelle et celle qui serait nécessaire.

**Méthode:**

À l’aide d’un sondage en ligne à méthodes mixtes, nous avons demandé à un échantillon de 378 personnes exerçant divers rôles en santé publique dans diverses régions du Canada d’évaluer leur compétence en communication actuelle et leur accord avec un projet de cadre de compétence en communication modernisé, fondé sur les données probantes. Le sondage a été diffusé dans les deux langues officielles par des organismes partenaires et dans les médias sociaux. Nous avons effectué une analyse statistique descriptive pour évaluer le niveau d’accord, et nous avons analysé les écarts par rapport aux rôles et à l’expérience en santé publique.

**Résultats:**

La plupart des personnes participantes se sont dites compétentes en communication, mais certains aspects ont obtenu des notes plus faibles. Les 21 énoncés de compétences proposés ont été bien acceptés, avec quelques écarts observés entre le savoir-faire et les niveaux d’expérience. La demande de formation en communication est élevée.

**Conclusion:**

L’accord général avec les énoncés est signe de l’appui à un cadre de compétence en communication modernisé chez le personnel professionnel de notre échantillon. Une étude est en cours pour réunir d’autres éléments probants sur les demandes de la main-d’œuvre en santé publique en matière de communication et sur les écarts observés dans l’accord général avec les énoncés proposés.

## Introduction

Canadian public health systems, while diversely structured and governed, are unified by their essential functions and the professional competencies necessary to achieve these functions (Public Health Agency of Canada, 2021). In 2008, the Public Health Agency of Canada (PHAC) published Core Competencies for Public Health in Canada, a framework of 36 competency statements organized into seven categories including communication (Public Health Agency of Canada, 2008). These competency statements serve as a guide for public health education (including Master of Public Health programs), recruitment, professional training, and maintenance of a skilled and proficient workforce (Public Health Agency of Canada, 2007, 2008). Numerous examples of competency frameworks exist across health professions (including but not limited to physicians, public health professionals, and nurses), and have been effectively used to define roles and guide training, professional development, and human resource management (Batt et al., 2020). Discipline-specific competency frameworks (e.g., epidemiology, communication, leadership) build on the Core Competencies to allow for additional mastery.

Outside of Canada, other jurisdictions including the United States (Council on Linkages Between Academia & Public Health Practice, 2021) and the European Union (World Health Organization & Regional Office for Europe, 2020) have their own public health competency frameworks. Each framework similarly outlines essential knowledge, skills, attitudes, and behaviours for public health practice. Public health competency frameworks have integrated communication into the competency statements, and many have a communication domain.

Communication is an integral part of public health research and practice and the PHAC Core Competencies include four competencies within the communication category (Table 1) (Public Health Agency of Canada, 2008). Public health communication consists of both internal and external communication such as communication to partners, communication to the public, and communication within governments/organizations (Bernhardt, 2004; Public Health Agency of Canada, 2008). Intersecting all areas and roles in the field, public health communication blends elements of various disciplines including science, social science, and marketing, and includes general communication as well as health communication which more specifically focuses on informing people about health information to achieve health goals (Bernhardt, 2004). Public health is bidirectionally involved in communication through both the transmission and receiving of information such as health promotion campaigns and seeking community feedback or performing evaluations, respectively (Rimal & Lapinski, 2009). Public health practitioners must possess foundational communication competence and highly advanced communication skills and knowledge to effectively manage challenging communication environments, especially during crises (Centers for Disease Control & Prevention, 2018).

**Table 1:** PHAC communication Core Competency statements (Public Health Agency of Canada, 2008) and short reference names

Competency short name	Competency statement
Communication skills	6.1 Communicate effectively with individuals, families, groups, communities, and colleagues
Interpret information	6.2 Interpret information for professional, non-professional, and community audiences
Mobilize people	6.3 Mobilize individuals and communities by using appropriate media, community resources, and social marketing techniques
Current technology	6.4 Use current technology to communicate effectively

The COVID-19 pandemic has been a salient reminder of the importance of communication in public health as existing and emerging communication challenges have been further emphasized. Challenges during the pandemic have included maintaining communication transparency and appropriate message timing, use of plain language, and communication consistency across public health systems within Canada (Lowe et al., 2022). The Chief Public Health Officer of Canada reflected on these challenges in a 2021 report, highlighting priorities for public health in Canada including clear, coherent, and transparent communication tailored to various communities, including equity-deserving communities (Public Health Agency of Canada, 2021).

The PHAC Core Competencies were intended to evolve alongside public health challenges and demands through ongoing revision; however, no updated framework has been published since the release in 2008 (Public Health Agency of Canada, 2008). Modernization of the PHAC Core Competencies is now underway with PHAC commissioning the National Collaborating Centres for Public Health (NCCPH) to lead this work. The age of the current competency framework combined with challenges experienced during the COVID-19 pandemic has prompted multiple calls for updated competency statements, especially those focused on communication. Specifically, the National Collaborating Centre for Indigenous Health (NCCIH) has called for updated Core Competencies which better reflect the importance of culturally safe communication, among other recommendations (Hunt, 2015). As part of the goal to build workforce expertise and capacity, the Chief Public Health Officer suggested the Core Competencies should be modernized to better address emerging public health requirements and responsibilities, including communication tasks such as risk communication, addressing mis/disinformation, and knowledge translation/mobilization (Public Health Agency of Canada, 2021). In 2022, the Canadian Public Health Association (CPHA) provided recommendations for strengthening the Canadian public health system, including a recommendation to update and strengthen competency statements and align training opportunities to address topics such as risk communication and community engagement (Canadian Public Health Association, 2022).

Given these direct calls for updated communication competency statements and the vital role communication plays in the success of public health, it is pertinent that we have a strong understanding of the communication skills, knowledge, attitudes, and behaviours which are critical to the current and future public health workforce. To achieve the ambitious objectives and functions of public health, and to address complex public health challenges, a communication competency framework that is modern and comprehensive is necessary to guide workforce planning and development and ensure effective public health action.

Despite the importance of public health communication competencies, there exists little literature on the topic. Some of the limited recommendations for competency framework development from the literature include developing competency frameworks that are justified by supporting literature, and consulting content experts and competency framework end-users (such as the relevant workforce) to build consensus (Batt et al., 2020).

Given the pressing need for and lack of literature on updated public health communication competency statements, the aim of this research is to gather evidence from a sample of public health practitioners and researchers about their communication competence and perceived importance of communication knowledge, skills, values, and behaviours for effective and precise public health practice. This research is part of an iterative consensus-building project to develop a public health communication competency framework for Canada. The objectives of this research are to:

1. Measure self-reported communication competencies of respondents based on current communication competency statements.
2. Measure the respondents’ level of agreement with proposed communication competency statements.
3. Explore potential differences in agreement with proposed competency statements due to communication focus of the respondent’s role and their job experience.
4. Explore the public health workforce’s current communication training level and interest in further training opportunities.

## Methods

### Ethics

This research was approved by the University of Guelph Research Ethics Board REB#23–04-004.

### Development of the public health communication competency statements

A draft list of communication competency statements was developed by first identifying published frameworks from various countries (e.g., Canada, USA, New Zealand), associations (e.g., Australian Health Promotion Association, National Commission for Health Education Credentialing, Council of Academic Public Health Institutions Australia), and the literature (Park et al., 2021) (see Online Resource 1, Supplementary Table 1 for a full list of frameworks). Next, 15 health communication textbooks were procured, and the table of contents, glossaries, and chapters were examined. Three researchers with expertise in health communication (JEM, MM, and DM) collaboratively built an Excel (Microsoft Excel, 2018) spreadsheet adding expert knowledge and all relevant key terms and concepts found in existing communication competency statements, textbooks, and the literature, and iteratively distilling them into competency statements by combining like terms and removing redundancy during three separate meetings. An initial list of competency statements (Table 2) was developed based on the key areas, and the terms and concepts identified within each, and was revised in consultation with the research team.

**Table 2:** Draft communication competency statements for public health in Canada

Statement ID	Competency statement
C1	Apply key relevant interdisciplinary theories, frameworks, toolkits, and best practice guidelines to develop health communication initiatives
C2	Ground health communication efforts in appropriate philosophical and critical perspectives, including social and health justice, health equity, and public health ethics
C3	Foster attitudes and approaches for fair, equitable, and inclusive communication (e.g., empathy, respect, solidarity, compassion, reciprocity, and reflexivity)
C4	Use an audience-centered, participatory approach to develop communication initiatives (e.g., co-design, community-based approaches, and two-way communication)
C5	Identify and integrate factors that influence how different audiences find, understand, use, apply, and act on health information
C6	Apply the necessary knowledge synthesis and research skills to develop evidence-informed, theory-based health communication initiatives
C7	Select and apply appropriate approaches and tools for the design of health messages to ensure they are accurate, clear, accessible, credible, and understandable
C8	Design health communication initiatives that inform and/or persuade to impact knowledge, attitudes, intentions, behaviours, self-efficacy, resilience, and individual and population health
C9	Design health messaging that is transparent, trustworthy, tailored, and, where applicable, delivered by an appropriate messenger or source
C10	Identify and use current and emerging communication channels, settings, and technologies to meet audience information needs
C11	Monitor information ecosystems to identify and address related challenges (e.g., information-seeking behaviours, information availability and accessibility, and mis/disinformation)
C12	Plan evidence-based communication initiatives with clear goals and objectives, appropriate timing, partnerships, and resources that meet community needs
C13	Identify audiences and generate nuanced audience profiles (e.g., type, level, segmentation) to guide communication
C14	Implement the appropriate approaches for communication initiatives based on context and audience characteristics
C15	Plan and implement appropriate evaluation of communication initiatives
C16	Communicate in a culturally competent and safe manner in a way that is informed by the relationship between culture, language, and health
C17	Attend carefully to the barriers to and the unintended consequences of impactful health communication, which may limit effectiveness and exacerbate harms and health disparities
C18	Effectively apply communication skills in activism, advocacy, and partnerships to improve individual health, population health, and health equity
C19	Mobilize evidence and different ways of knowing to inform policy and practice decisions in public health
C20	Use specialized communication (e.g., crisis, risk, clinical) to interpret, translate, and tailor complex information to guide action
C21	Create and use a range of communication materials (e.g., policy brief, news media article, presentations, recordings, stories) and types (e.g., oral, written, visual) that meet audience needs and reflect their values

### Survey design

Recruitment materials and the survey were available in English and French. The survey was comprised of four sections: (1) demographic information, (2) self-reported competence in the communication-related Core Competencies for Public Health in Canada (Public Health Agency of Canada, 2008), (3) self-reported competence in communication channels, audiences, and types, and (4) the agreement with each draft communication competency statement (Table 2).

Section 1 of the survey asked about job title, length of time in current role, gender, education, and whether their role was communication-focused, and about their organization (type, province or territory location, priority population served).

Sections 2 and 3 examined self-reported proficiency in communication Core Competencies (Table 1) and use of communication types, channels, and audiences identified in the competency statements, respectively. Participants rated their competence in communication on a 4-point Likert-type scale from *Not Competent* to *Very Competent*. A 4-point scale was used rather than a 5-point scale to omit the neutral midpoint which can be difficult to interpret (Chyung et al., 2017).

Section 4 assessed participants’ agreement with each of the proposed communication competency statements in Table 2 on a 4-point Likert scale from *Strongly Disagree* to *Strongly Agree* and asked for suggestions for additional competency statements. Consensus was defined a priori as at least 75% of participants rating each statement as *Agree* or *Strongly Agree*, which was the median threshold found in a systematic review of consensus-building studies (Diamond et al., 2014)*.* Statements which receive consensus among survey participants will be included in a final consensus-building process to arrive at a final framework. Participants were also asked about their views on the adequacy of health communication training and education in Canada and their interest in health communication professional development opportunities.

### Survey procedure

The survey (Online Resource 2) was available online via Qualtrics (Qualtrics, 2020) from June 20 to July 28, 2023. Individuals were eligible to participate if they were 18 years and older and currently worked in public health in Canada. To reach participants, the survey was shared through social media accounts (LinkedIn and Twitter) of the research team and partner organizations, including CPHA, National Collaborating Centres for Public Health (NCCPH), and provincial public health associations; e-newsletters and listservs of relevant organizations, including CPHA, Ontario Veterinary College, NCCPH, the Canadian Institute of Public Health Inspectors, provincial public health associations, provincial departments of health, and the Indigenous Physicians Association of Canada; and recruitment emails from the research team. Participants who completed the survey were encouraged to share it among their networks.

### Analysis

Descriptive statistics were performed using R statistical software (R Core Team, 2022) and Excel (Microsoft Excel, 2018) to identify participant characteristics, self-reported communication competence, and agreement with the draft list of health communication competency statements, and identify areas for further analysis within the next phases of the consensus-building process related to developing a health communication competency framework (namely key informant interviews and a modified Delphi technique). Strong agreement with proposed competency statements was stratified according to two demographic variables (communication-focused roles, and time in role) and an upper quartile cutoff was used to identify competency statements with higher between-group variation in *Strong Agreement*.

Partial response data were carefully evaluated for evidence that the survey was completed; all participant responses were retained for analysis. Demographics of participants providing partial responses were compared to demographics of those providing complete responses. There were no notable demographic differences or trends in partial responses.

Suggestions for additional competency statements and further comments captured in responses to open-ended questions were thematically analyzed and the top three themes most commented on by participants were reported on. First, MM coded the data in NVivo 12 Plus (Lumivero, 2020) where it overlapped among participants or provided important suggestions and comments. Next, the codes were organized into themes and discussed and refined among the research team and descriptive quotes were captured.

## Results

### Modernized public health communication competencies

Our iterative process of distilling key health communication terms, concepts, and competencies resulted in a list of 21 proposed public health communication competency statements (Table 2) which participants provided feedback on in the survey.

### Participant characteristics

A total of 378 participants responded to the survey and 82% of those participants completed the survey in full, with another 11% skipping no more than one question (Table 3). The vast majority responded in English (97%), identified as women (85%), and were employed by an organization that identifies Indigenous Peoples as a priority population (92%). Most respondents completed graduate education (54%), worked in a local public health unit or regional health authority (54%), and worked in Ontario (61%). Beyond the organizations listed in Table 3, other employers identified by participants included healthcare, not-for-profit, and Indigenous organizations. Most commonly (38%), respondents had worked between 1 and 5 years in their current role.

**Table 3:** Demographics of survey participants (*n* = 378) and their organizations/employers

Personal variable/characteristic	*n* (%)†
Language of survey completion	
English	367 (97%)
French	11 (3%)
Time in current role	
1–5 years	143 (38%)
More than 10 years	112 (30%)
Less than 1 year	75 (20%)
6–10 years	48 (13%)
Gender	
Woman (includes everyone who identifies as a woman)	321 (85%)
Man (includes everyone who identifies as a man)	39 (10%)
Agender	3 (1%)
Non-binary	2 (1%)
Gender queer	1 (0.3%)
Gender fluid	0
Prefer to self describe	0
Choose not to respond	12 (3%)
Education	
Completed graduate education	203 (54%)
Completed college/university	109 (29%)
Professional degrees	36 (10%)
Some graduate education	27 (7%)
Some college/university	3 (1%)
High school education	0
Organization variable/characteristic	*n* (%)†
Organization type	
Local public health unit/regional health authority	204 (54%)
Provincial/territorial government/agency	69 (18%)
Federal government/agency	55 (15%)
Municipal government	14 (4%)
Non-governmental organization	12 (3%)
Academia	11 (3%)
Community health centre	3 (1%)
Private industry	1 (0.3%)
Other; please specify:	7 (2%)
Choose not to respond	2 (1%)
Region*	
Ontario	231 (61%)
National (Canada)	39 (10%)
British Columbia	31 (8%)
Alberta	30 (8%)
Quebec	14 (4%)
Manitoba	10 (3%)
Saskatchewan	10 (3%)
Nova Scotia	7 (2%)
Prince Edward Island	5 (1%)
Yukon Territory	5 (1%)
New Brunswick	4 (1%)
Newfoundland and Labrador	4 (1%)
Nunavut	3 (1%)
Northwest Territories	2 (1%)
Choose not to respond	5 (1%)
Organization identifies Indigenous peoples/communities as a priority population	
Yes	347 (92%)
No	31 (8%)

*Multiple categories were possible; category totals may exceed 100%

†Percentages were rounded to the nearest whole number; category totals may exceed 100%

**Fig. 1:**
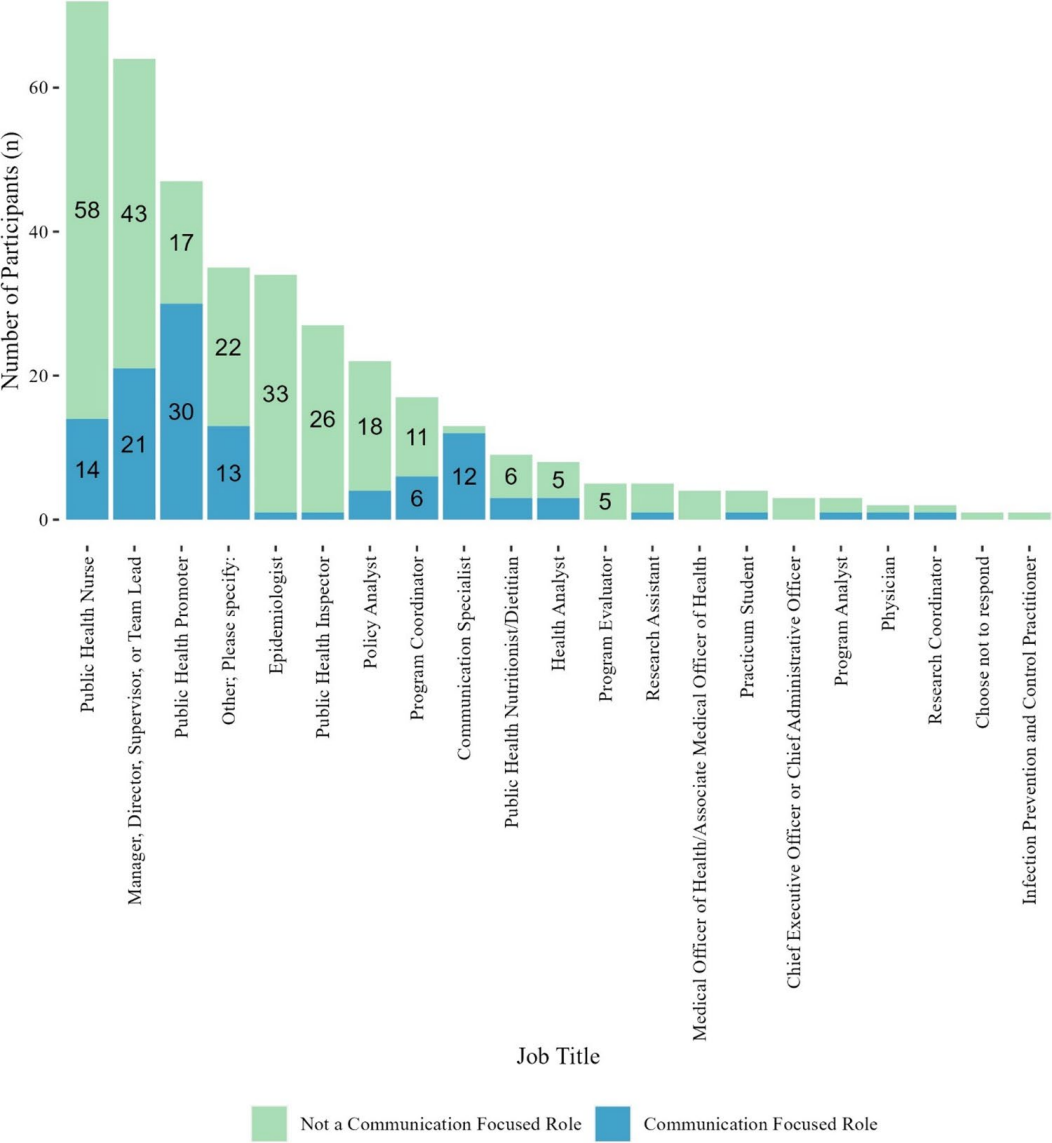
Participant job titles and self-reported communication focus of the role (*n* = 378) (see Online Resource 1, Supplementary Table 2 for a complete breakdown of Job Titles and Communication Roles with Frequencies). Data labels represent the number of participants, omitting labels for counts less than five for clarity

Public health nurse was the most reported job title (19%; of whom, 19% reported having a communication focus and 81% reported no communication focus), followed by a leadership role (team lead, supervisor, manager, director) (17%; of whom, 33% reported having a communication focus and 67% reported no communication focus), and public health promoter (12%; of whom, 64% reported having a communication focus and 36% reported no communication focus) (Fig. 1). Other job titles reported included program assistants (*n* = 7; 2%), knowledge mobilization practitioners (*n* = 5; 1%), and environmental public health practitioners (*n* = 3; 1%).

**Fig. 2:**
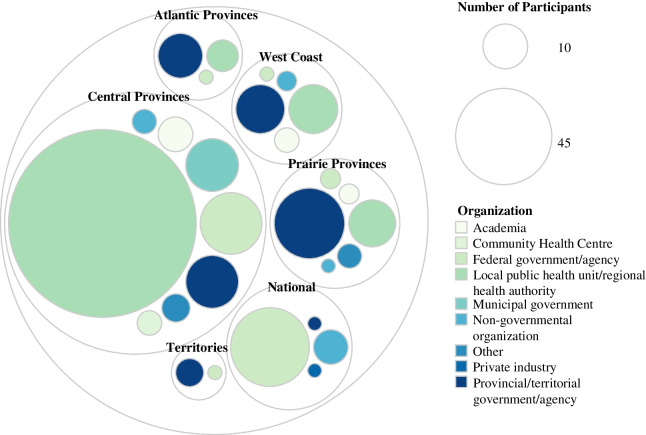
Participant organization and region (*n* = 373; five non-responses omitted). West Coast = British Columbia; Prairie provinces = Alberta, Saskatchewan, and Manitoba; central provinces = Ontario and Quebec; Atlantic provinces = New Brunswick, Prince Edward Island, Nova Scotia, and Newfoundland and Labrador; Territories = Yukon, Northwest Territories, and Nunavut

Figure 2 depicts the number of participants within the various types of organizations by region. Within the central provinces (Ontario and Quebec), most participants were employed by a local public health unit or regional health authority, followed by a federal government agency. Participants within the Prairies (Alberta, Saskatchewan, and Manitoba), Atlantic Canada (New Brunswick, Prince Edward Island, Nova Scotia, and Newfoundland and Labrador), and the territories (Yukon, Northwest Territories, and Nunavut) were mainly employed by provincial governmental agencies, while those in British Columbia were mainly employed by both provincial governmental agencies and regional health authorities. Nationally, participants worked within federal governmental agencies.

### Self-reported competence

Participants self-reported a high rate of overall communication competence (*Competent* or *Very Competent*; *n* = 296, 78%) (Table 4). Across the four Core Competencies related to communication, most participants reported being *Competent* or *Very Competent*, except for mobilizing people (*n* = 172, 46%). When broken down by communication types, audiences, and channels, most participants also reported being *Competent* or *Very Competent* except for web 2.0–related channels (website/blog maintenance and creation; *n* = 126, 33%, and software/apps; *n* = 127, 34%) and news media (*n* = 127, 34%).

**Table 4:** Self-reported communication competence of Canadian public health professionals (*n* = 378)

Variable	No response *n* (%)†	Not competent *n* (%)†	Somewhat competent *n* (%)†	Competent *n* (%)†	Very competent *n* (%)†
Self-reported overall competence	6 (1.6%)	2 (0.5%)	74 (19.6%)	228 (60.3%)	68 (18.0%)
PHAC core competency					
Interpreting information	1 (0.3%)	2 (0.5%)	39 (10.3%)	191 (50.5%)	145 (38.4%)
Communication skills	1 (0.3%)	4 (1.1%)	35 (9.3%)	205 (54.2%)	133 (35.2%)
Current technology	1 (0.3%)	19 (5.0%)	115 (30.4%)	179 (47.4%)	64 (16.9%)
Mobilize people	1 (0.3%)	54 (14.3%)	151 (39.9%)	136 (36.0%)	36 (9.5%)
Communication types					
Written	1 (0.3%)	2 (0.5%)	30 (7.9%)	165 (43.7%)	180 (47.6%)
Verbal/oral	3 (0.8%)	3 (0.8%)	37 (9.8%)	195 (51.6%)	140 (37.0%)
Visual	2 (0.5%)	36 (9.5%)	129 (34.1%)	164 (43.4%)	47 (12.4%)
Communication audiences					
Professional-public health	2 (0.5%)	7 (1.9%)	44 (11.6%)	181 (47.9%)	144 (38.1%)
Public	2 (0.5%)	8 (2.1%)	73 (19.3%)	194 (51.3%)	101 (26.7%)
Partner	3 (0.8%)	7 (1.9%)	78 (20.6%)	207 (54.8%)	83 (22.0%)
Professional-non-public health	3 (0.8%)	57 (15.1%)	136 (36.0%)	125 (33.1%)	57 (15.1%)
Communication channels					
E-mail	3 (0.8%)	0	12 (3.2%)	148 (39.2%)	215 (56.9%)
Virtual meetings	6 (1.6%)	3 (0.8%)	25 (6.6%)	161 (426%)	183 (48.4%)
In-person	7 (1.9%)	1 (0.3%)	23 (6.1%)	165 (43.7%)	182 (48.1%)
Reports/documents	7 (1.9%)	9 (2.4%)	43 (11.4%)	145 (38.4%)	174 (46.0%)
Telephone	3 (0.8%)	2 (0.5%)	37 (9.8%)	177 (46.8%)	159 (42.1%)
Presentations	4 (1.1%)	5 (1.3%)	56 (14.8%)	178 (47.1%)	135 (35.7%)
Webinars	5 (1.3%)	33 (8.7%)	109 (28.8%)	153 (40.5%)	78 (20.6%)
Conferences	5 (1.3%)	29 (7.7%)	101 (26.7%)	168 (44.4%)	75 (19.8%)
Online resources/websites	8 (2.1%)	43 (11.4%)	112 (29.6%)	151 (39.9%)	64 (16.9%)
Social media	7 (1.9%)	57 (15.1%)	140 (37.0%)	111 (29.4%)	63 (16.7%)
Community networks	8 (2.1%)	44 (11.6%)	127 (33.6%)	142 (37.6%)	57 (15.1%)
Website/blog maintenance/creation	3 (0.8%)	125 (33.1%)	124 (32.8%)	87 (23.0%)	39 (10.3%)
News media	6 (1.6%)	112 (29.6%)	133 (35.2%)	91 (24.1%)	36 (9.5%)
Software/apps	6 (1.6%)	120 (31.7%)	125 (33.1%)	98 (25.9%)	29 (7.7%)

†Percentages were rounded to one decimal place; category totals may exceed 100%

### Competency statement agreement

Overall, participants had very high agreement (*Agree* or *Strongly Agree*; average of 92%), with all proposed competency statements meeting the a priori threshold (75% *Agree or Strongly Agree*) for inclusion in the next round of the consensus-building process (Table 5). The upper quartile for *Strongly Agree* was 60% (C9, C16, C7, C12, C21, C19) and the lower quartile was 50% (C13, C11, C1, C4, C8, C20, C18). The median for *Strongly Agree* was 92.6% and the interquartile range was 10.6%.

**Table 5:** Workforce agreement with proposed communication competency statements (*n* = 378)*

Proposed statement	No response *n* (%)†	Strongly disagree *n* (%)†	Disagree *n* (%)†	Agree *n* (%)†	Strongly agree *n* (%)†
C1	6 (1.6%)	2 (0.5%)	28 (7.4%)	167 (44.2%)	175 (46.3%)
C2	9 (2.4%)	2 (0.5%)	20 (5.3%)	139 (36.8%)	208 (55.0%)
C3	8 (2.1%)	5 (1.3%)	14 (3.7%)	134 (35.4%)	217 (57.4%)
C4	6 (1.6%)	4 (1.1%)	18 (4.8%)	165 (43.7%)	185 (48.9%)
C5	6 (1.6%)	3 (0.8%)	17 (4.5%)	142 (37.6%)	210 (55.6%)
C6	5 (1.3%)	4 (1.1%)	25 (6.6%)	138 (36.5%)	206 (54.5%)
C7	6 (1.6%)	3 (0.8%)	6 (1.6%)	116 (30.7%)	247 (65.3%)
C8	7 (1.9%)	6 (1.6%)	25 (6.6%)	154 (40.7%)	186 (49.2%)
C9	6 (1.6%)	3 (0.8%)	7 (1.9%)	90 (23.8%)	272 (72.0%)
C10	7 (1.9%)	4 (1.1%)	18 (4.8%)	138 (36.5%)	211 (55.8%)
C11	6 (1.6%)	6 (1.6%)	32 (8.5%)	185 (48.9%)	149 (39.4%)
C12	7 (1.9%)	3 (0.8%)	5 (1.3%)	123 (32.5%)	240 (63.5%)
C13	7 (1.9%)	9 (2.4%)	37 (9.8%)	189 (50.0%)	136 (36.0%)
C14	7 (1.9%)	2 (0.5%)	10 (2.6%)	158 (41.8%)	201 (53.2%)
C15	8 (2.1%)	5 (1.3%)	9 (2.4%)	146 (38.6%)	210 (55.6%)
C16	7 (1.9%)	3 (0.8%)	8 (2.1%)	94 (24.9%)	266 (70.4%)
C17	10 (2.6%)	3 (0.8%)	19 (5.0%)	123 (32.5%)	223 (59.0%)
C18	7 (1.9%)	5 (1.3%)	26 (6.9%)	152 (40.2%)	188 (49.7%)
C19	7 (1.9%)	5 (1.3%)	13 (3.4%)	125 (33.1%)	228 (60.3%)
C20	9 (2.4%)	4 (1.1%)	28 (7.4%)	149 (39.4%)	188 (49.7%)
C21	7 (1.9%)	5 (1.3%)	11 (2.9%)	126 (33.3%)	229 (60.6%)

*See Table 2 for list of competency statements

†Percentages were rounded to one decimal place; category totals may exceed 100%

**Fig. 3:**
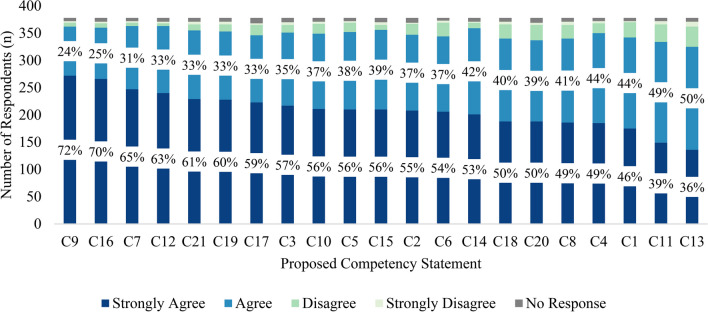
Workforce agreement with proposed communication competency statements (*n* = 378) (see Table 2 for the list of competency statements). Stacked columns represent the proportion of participants who responded according to each Likert-type response option. Data labels represent the percentage of participants who responded *Strongly Agree* and *Agree*, respectively, to each proposed competency statement

Figure 3 depicts participants’ rating of *Strongly Agree* with each competency statement in descending order. Statements related to trusted and tailored messages (C9; 72%) and culturally competent communication (C16; 70%) had the highest *Strongly Agree* rating. Statements related to information ecosystems (C11; 39%) and audience segmentation/needs assessment (C11; 36%) were the two statements with the lowest *Strongly Agree* rating.

### Stratification of competency statement agreement

When agreement with the competency statements was compared between those who reported being in communication-focused roles versus those who reported not having a communication-focused role, group differences from the *Strongly Agree* category were found for some statements (Online Resource 1, Supplementary Table 3). Descriptive group differences were found for competency statements describing audience segmentation/needs assessment (C5 and C13), designing health communication campaigns (C8 and C14), using specialized communication (C20), and communication types (C21). A higher percentage of respondents in communication-focused roles strongly agreed with these statements compared to those in non-communication-focused roles.

When agreement was compared between participants who reported being in their current role between 1 and 5 years and those who have been in their role for 6 years or more, group differences for the *Strongly Agree* category were also found (Online Resource 1, Supplementary Table 4). Descriptive group differences were found with participants who had been in their role for longer than 5 years providing stronger agreement for competency statements related to applying specialized communication skills (C18) and health literacy (C7), compared to participants who had been in their role for 5 years or less. Participants who had been in their role for longer than 5 years provided less agreement for competency statements related to information ecosystems (C11), designing communication campaigns (C14), and communication types (C21), compared to participants who had been in their role for 5 years or less.

### Suggestions for additional competency statements

Participants suggested additional competencies for inclusion and areas of emphasis within existing competencies. Table 6 lists themes generated from participants’ recommendations and corresponding illustrative quotes. While many of the suggestions were represented in the list of proposed competency statements, the themes highlight areas that may need to be strengthened and will thus be included in the discussions during the next phases of the research process (i.e., key informant interviews and modified Delphi technique) to develop the final list of competency statements.

**Table 6:** Themes for additional communication competencies suggested by participants

Themes (additional competencies)	Selected quote(s)
Timeliness	“Not sure if timeliness was mentioned. Information is fast-paced, and we are always lagging.”
Plain/clear language	“…explain scientific or public health evidence in a simple manner that maintains accuracy.”
Social listening	“…social listening, online and offline information gathered to prepare recommendations and help initiate co-creation of programs and communication about a public health issue.”
Co-design with equity-deserving groups	“Strengths-based approach when speaking about people who are involved in certain risks (e.g., drug use, sex work, etc.) needs to be incorporated too.” “I…am surprised by the lack of awareness of basic antiracist and social justice theory within public health. Learning about the impact of colonialism is also critical.” “…varied modes of communication appropriate for the community (e.g., storytelling, working with Elders—Afro-centric and Indigenous communities).”
Cultural safety/humility	“Communication with a high degree of cultural safety and humility.”
Shift to systems level (from individual/behaviour change)	“I think that public health is supremely challenged right now because it remains focused on individual behaviour change.” “… intersectionality… that’s a pretty essential part of understanding health and the social determinants of health.”
Leverage other disciplines	“There is a whole body of education on effective marketing as seen on mainstream media and yet we don’t leverage similar knowledge to inform our marketing of ideas.”
Facilitation skills	“… facilitation skills—facilitating meetings, facilitating community networks, communication for relationship/partnership building.”
Communicating with internal audiences	“Consider competencies critical for internal communication in a skilled, evidence/data-driven, and effectively managed and led public health workforce.”
Macro- and micro-level policies	“Cultural competency training is so important and needs to be reflected in the communication policies.” “Creating policies… that support organizations, agencies, or health centres to ensure people can equitably access, understand, and use health information.”

Participants were also asked to provide any additional comments in an open-ended question. Themes focusing on areas for action emerged (Table 7) with some related to governance of the competencies and effective implementation, and others to the need for training and competency mastery to impact public health.

**Table 7:** Themes for areas of action related to communication competencies as suggested by participants

Themes (areas for action)	Selected quote(s)
Need for formalized health communication training	“Learning opportunities to foster the development and maintenance of these competencies among public health practitioners will be essential.” “This [health communication] is an area where I have been frustrated with the lack of training and knowledge amongst practitioners.” “These [draft health communication competencies] should be built into basic nursing, epidemiology, nutrition, and health inspection courses so that all public health practitioners will start out with at least the basic communication competency skills.”
Need for core and discipline-specific health communication competencies	“All staff need basic principals [*sic*] and knowledge but that does not mean we do not need specialized health marketing/communications staff.” “There is often a tension within our organization between our communications department and our population health department. I do think there is likely a need for a competency related to navigating the communications landscape internally—advocating for a role for Public Health professional on the communications team, for example.” “‘Can we do it all?’ We have a definitive need for more training but … need further specialists in communication and multidisciplinary teams.”
Need for accessible and actionable health communication framework	**“…** issue isn’t changing the competencies; it’s doing a better job of sharing the competencies and building a culture where all public health professionals see this as a crucial part of their work.” “I’d like to see examples of the different types of communication activities that different roles/public health practitioners would do.”
Need for coordination and resources shared across public health jurisdictions	“Communications for Public Health should be centralized for each province… Consolidation would allow for specialized staff and more consistent, effective and accurate information.” “In Ontario-we have individual health units promoting things in different ways we should all be one united front to be equitable across Ontario. As a rural health unit, we don’t have the resources that bigger health units do to communicate in an effective manner with the public.”
Need for increased focus on health equity and social justice	“We must not continue to add to these disparities [unhoused, lack of transportation, etc.] by requiring an email address or health card # to book appointments, etc.” “Not enough focus on equity data.”
Need for inclusive languages and practices for competency development	“…labelling certain groups as “vulnerable” forces us to view them as such, when in fact they are stronger and more resilient than most, however our language forces us to view them as vulnerable when in fact our systemic/structural inequities and discrimination create this “vulnerability”.” “…focus on how we build healthy relationships with each other, we will ultimately develop safe and supportive environments where everyone has a sense of accountability and belonging.”

### Training in communication and health communication

Most participants (86%) reported there are inadequate health communication training opportunities in Canada (Supplementary Table 5). There was strong interest in additional training opportunities, with webinars (84%), an online micro-credential (81%), and digital resources (63%, 62%) being of particular interest. Most participants reported receiving informal communication (63%) and health communication (55%) training (e.g., on-the-job training, self-taught, webinars), relative to formal training in communication (40%) and health communication (37%) (e.g., certificates, undergraduate or master’s degrees). Nearly 30% reported no training whatsoever in communication or health communication. Formal health communication training included masters-level training (e.g., Master of Public Health), courses (e.g., PHAC Skills Enhancement), and undergraduate-level training (e.g., Nursing).

## Discussion

Canadian public health is reliant on Core Competencies to ensure professionals in the sector are equipped to address the evolving health needs of the population. Our study’s findings indicate self-reported gaps in communication competence in areas such as digital technologies, visual communication, and mobilizing people. Additionally, participants had high levels of agreement with the 21 newly proposed competency statements, supporting the need for a modernized public health communication competency framework. Furthermore, participants reported a substantial gap in communication and health communication training in the Canadian public health workforce.

These findings underscore the critical importance of building competencies for effective communication to address the evolving health needs of the population. The ongoing revision of the PHAC Core Competencies by the NCCPH is a timely endeavour, and this study contributes essential data to inform the development of a comprehensive public health communication competency framework for Canada. Our research team is collaborating with the NCCPH Core Competency Project Team to support and align the timelines of these parallel projects. Our framework will be a companion document outlining the foundational and discipline-specific competencies required for Public Health in Canada. This study collected evidence on current communication competence and agreement with proposed communication competency statements from a sample of the national public health workforce (*n* = 378). This study is part of a larger multi-step consensus-building project to develop a public health communication competency framework for Canada.

### Self-reported communication core competencies varied

Most participants (78%) reported overall communication competence (*Competent* or *Very Competent*); however, self-reported communication competence was far more variable for specific communication Core Competencies. Self-reported competence for using current technology and mobilizing people was considerably lower. Similar trends were observed in specific communication types, audiences, and channels, with visual communication, communicating with non-public health audiences (e.g., news, politicians), and using new media (e.g., blogs, social media). Across all sectors related to health, understanding, applying, and adapting information communication technologies are increasingly important, although digital competence tends to be low and workforce development is needed (Shiferaw et al., 2020). A review of digital competency frameworks for health found 28 digital health competency domains, including digital communication, ethics, data privacy, and security, among others, that should be incorporated into workforce training and development (Nazeha et al., 2020). Further, mobilizing people builds community capacity, social justice, and positive health outcomes through participatory action and co-production, which requires a range of diverse and integrated competencies (Vargas et al., 2022). Strengthened and modernized competencies and related workforce development in these areas are needed to address these modern and complex public health issues and opportunities. This is supported by the recent advocacy campaign by the Canadian Public Health Association to produce an updated list of public health competencies and produce national training opportunities based on modern competencies (Canadian Public Health Association, 2022).

This area of opportunity for training and strengthened competency statements is also reflected by current training rates and perceived need for and interest in training. Almost one third of participants reported no training in communication generally, and in health communication specifically. The vast majority also reported that existing health communication training opportunities were inadequate. A previous step in this multi-step consensus-building process found professional development opportunities for public health communication in Canada are unorganized and do not comprehensively address communication competencies (MacKay et al., 2024). We also found Canadian public health graduates may not be receiving sufficient public health communication training to develop competencies and meet the demands of the current public health landscape (MacKay et al., 2023). Overall, these results together highlight the need for comprehensive professional development and student training that reflects modern public health communication competencies.

### High agreement with proposed public health communication competency statements

The proposed 21 communication competency statements (Table 2) were widely agreed upon by the participants with total agreement (*Agree* and *Strongly Agree*) ranging from 86% to 96%, meeting the a priori threshold for agreement. This indicates strong support for the proposed public health communication competency statements and meets recent calls for strengthened public health communication competencies (Canadian Public Health Association, 2022). This high level of agreement moves all 21 competency statements to the next stages of planned research involving key informant interviews and a modified Delphi technique where the proposed statements will be revised, forming a final list that receives consensus regarding the content, wording, and length.

The upper quartile of *Strongly Agree* included competency statements relating to message content, cultural competence in communication, effective planning of communication, using a range of communication materials and types, and mobilizing evidence to inform decisions indicating particularly strong support among the workforce for these statements. Notably, the statement regarding cultural competence and safety in communication was the second most strongly agreed upon and further emphasized as important in open-ended feedback provided by some participants. This echoes key recommendations on updating public health competency frameworks to better address cultural safety, especially for Indigenous populations, made by the NCCIH (Hunt, 2015).

### Differences in agreement by communication-focused roles and length of time in current role

Given the diversity of positions that exist within public health, we examined whether a communication focus in roles impacted agreement with competency statements. Competency statements with the most between-group differences in strong agreement (upper quartile of > 17% difference between groups) were related to topics such as assessing and modifying communications for different audience profiles, specialized communications, and using a wide range of communication types and materials, in which the communication-focused roles had higher agreement than those in non-communication-focused roles (Online Resource 1, Supplementary Table 3). In contrast, statements with the least between-group differences related to topics such as communication theories, values and attitudes, public health philosophy, and foundations of communication such as planning, and synthesizing or producing evidence and using different communication channels, settings, and technologies (Online Resource 1, Supplementary Table 3).

There was also variation in how long respondents had been in their current role, recognizing that time in current role may not correlate to overall public health experience. Most participants had worked 5 years or less in their current role and some overlap with the most between-group differences and communication-focused roles were found. Namely developing communication campaigns and using different communication channels, settings, and technologies had overlapping group differences in the *Strongly Agree* category. Similarly, differences in level of competence and how important various skills were thought to be were also found in a study of primary care managers (Dikic et al., 2017).

The variation in agreement between roles and experience highlights areas for deeper discussion in the next steps of this research and indicates how expertise in public health communication may impact awareness and perceived importance of specialized skills. It also highlights the nuanced nature of public health communication competencies and emphasizes the importance of tailored training and development programs to address the specific needs of different roles within the public health workforce. Additionally, this variation may indicate a need for competency statements that are tailored to different experience and competence levels such as the Front Line, Management, and Senior Management tiers used in the US competency framework (Council on Linkages Between Academia & Public Health Practice, 2021).

### Foundational versus discipline-specific communication competencies

Variation in strength of agreement may indicate a distinction between what could be considered foundational versus discipline-specific communication competencies and thus offers valuable insights for the development of a robust competency framework. The lower quartile of *Strongly Agree* included competency statements related to nuanced audience profiles, information ecosystems, interdisciplinary theories, participatory approaches to communication, using communication to inform and persuade, using specialized communications, and using communication in activism/advocacy/partnerships. Compared to the upper quartile, which related to foundational elements of effective public health communication, the lower quartile may indicate competency statements that are more specialized and limited to specific roles, or require more advanced training. Although we did not explicitly ask about it, the potential utility of core and discipline-specific health communication competency frameworks was identified by multiple participants in their open-ended comments. Competency statements with high between-group variation, although meeting the threshold for a priori agreement and progression to the next stage of this research, will be evaluated in more depth to disentangle possible foundational versus discipline-specific competencies within the proposed public health communication framework.

This is consistent with other public health competency research and development which often identifies differences between specialist and non-specialist roles, on-the-job competency development, and division of responsibilities throughout organization structures (Bondy et al., 2008; Council on Linkages Between Academia & Public Health Practice, 2021). Foundational competencies are crosscutting integrated knowledge, skills, values, and behaviours for all individuals working in public health and provide the foundation for discipline-specific competency frameworks (de Beaumont Foundation, 2021). The combination of foundational competencies with discipline-specific skills allows for practitioners to effectively and impactfully address the changing and complex public health landscape.

### Strengths and limitations

To our knowledge, this is the first national survey sampling the public health workforce related to communication competence. The survey benefits from its ability to directly collect evidence from public health practitioners and researchers and is timely following the recent communication challenges faced by the sector during the COVID-19 pandemic (Public Health Agency of Canada, 2021) and ongoing renewal of the Core Competencies for Public Health in Canada (Public Health Agency of Canada, 2008).

The primary limitation of this study is the accessibility of the public health workforce and the representativeness of the survey sample. Distribution of the online survey was reliant on and limited by access to the public health workforce through partner organizations’ distribution channels (e.g., listservs, newsletters, mailing lists, websites) and social media. As these distribution channels have varying subscription and viewership numbers, there may be variability in reach and access to different sectors of the national public health workforce. We attempted to mitigate this limitation via broad distribution through professional associations, NCCPH, Indigenous organizations, and national networks to ensure a wide and representative distribution.

Although data are available for some public health occupations, in some provinces, there are no up-to-date data on the size, demographics, and professional composition of the national public health workforce. The best available information comes from a review of the public health workforce by Health Canada (2003) which reported that the national workforce consists of approximately 170 Medical Officers of Health or Associate Medical Officers of Health, 40 physicians hired by ministries, and approximately 12,000 public health nurses, with little to no information available on other public health professions (Health Canada, 2003). This same review estimated that public health nurses composed one third of the total workforce, indicating that the total workforce included approximately 36,000 employees (Health Canada, 2003). Given the limited data available and the age of that data, it is not possible to determine the representativeness of the study sample. Despite efforts by the Pan-Canadian Public Health Network, the lack of successful enumeration and characterization of the workforce is largely due to ambiguity as to what sector health professionals work in and aggregate reporting of health workforces (Public Health Agency of Canada, 2015; Smith et al., 2021). Given the value of workforce knowledge, future research is needed to comprehensively characterize the Canadian public health workforce.

The survey is also inherently limited by the question format. Although the survey used mixed methods to collect both quantitative and qualitative evidence, the closed-ended questions, while efficient at measuring competence and agreement, do not provide insight into the underlying reasoning or context for participants’ responses. The quantitative findings of this study would benefit from further qualitative research to provide more detailed insight, including rationales for levels of agreement and opinions of the proposed competency statements. The next steps of this consensus-building research will help to elucidate this.

## Conclusion

The high level of agreement among participants regarding the proposed communication competency statements is a strong endorsement of their relevance and applicability. This support signals a positive step towards modernizing and strengthening public health communication competency statements, aligning with recent calls for improvement by the Canadian Public Health Association and the Chief Public Health Officer of Canada. Variations in agreement between communication-focused roles, time in role, and when examining variation within the *Strongly Agree* category show a possible delineation of foundation and discipline-specific competencies that will be further explored. Regular revision of competency frameworks is critical to ensure they are responsive to the current public health landscape. This study has demonstrated areas of opportunity to improve the communication competence of the public health workforce, such as co-production and digital technologies. These findings form a strong foundation for a public health communication competency framework that can positively impact the public health workforce and form the foundation for future revisions. Further research should focus on gathering more evidence and building consensus on the specific communication demands of the public health workforce to best guide and support education, training, hiring/recruitment, and workforce development/management initiatives.

## Contributions to knowledge

What does this study add to existing knowledge?

- Describes current communication competence in a sample of the Canadian public health workforce.
- Identifies variation in self-reported communication competence.
- Demonstrates high agreement with proposed public health communication competency statements among the workforce sample.
- Highlights potential differences between foundational and discipline-specific or expert communication competencies.
- Addresses the lack of comprehensive evidence regarding Canadian public health communication competencies.
- Complements the wider renewal of the PHAC Core Competencies for Public Health and similar discipline-specific (e.g., public health leadership, digital public health, Black health) competency framework development underway.

What are the key implications for public health interventions, practice, or policy?

- There is a need for comprehensive public health communication training to bridge competency gaps and maintain a proficient workforce.
- Evidence supports existing calls for an updated public health communication competency framework in Canada to meet the complex needs of the public health workforce.
- Ongoing revision of competency frameworks and continuous re-evaluation of workforce communication competence will be needed to adapt to the evolving public health landscape and demands.

## Supplementary Information

Below is the link to the electronic supplementary material.

Supplementary file1 (DOCX 46 KB)

Supplementary file2 (DOCX 41 KB)

## Data Availability

Not applicable.
