# Supplementary material for: Modernizing public health communication competencies in Canada: A survey of the Canadian public health workforce
Source: Can J Public Health. 2024 May 16;115(4):664–79. doi: 10.17269/s41997-024-00890-w (PMC11303361; doi:10.17269/s41997-024-00890-w)
Supplement: Supplementary file 1 — Supplementary file1 (DOCX 46 KB) [file 41997_2024_890_MOESM1_ESM.docx]

**Online Resource 1**

**Supplementary Table 1 List of Competency Frameworks that Informed Competency Framework Development**

| **Competency Framework Name** | **Organization** | **URL** | **Communication-Related Competencies*** |
| --- | --- | --- | --- |
| **Core Competencies for Public Health in Canada 1.0** | Public Health Agency of Canada | https://www.canada.ca/content/dam/phac-aspc/documents/services/public-health-practice/skills-online/core-competencies-public-health-canada/cc-manual-eng090407.pdf | 6.1 Communicate effectively with individuals, families, groups, communities and colleagues.  6.2 Interpret information for professional, non-professional and community audiences.  6.3 Mobilize individuals and communities by using appropriate media, community resources, and social marketing techniques.  6.4 Use current technology to communicate effectively. |
| **The Pan-Canadian Health Promoter Competencies and Glossary** | Health Promotion Canada | https://www.healthpromotioncanada.ca/wp-content/uploads/2016/07/2015-HPComp-CompletePackage3-new-logo.pdf | 7.1 Provide information tailored to specific audiences (e.g., professional, community groups, general population) on population health status and health promotion action.  7.2 Apply communication methods and techniques to the development, implementation, and evaluation of health promotion action.  7.3 Use the media, information technologies, and community networks to receive and communicate information.  7.4 Communicate with diverse populations in a culturally-appropriate manner. |
| **ASPHER's European List of Core Competencies for the Public Health Professional** | Association of Schools of Public Health in the European Region (ASPHER) | https://journals.sagepub.com/doi/full/10.1177/1403494818797072 | D.1.10. Partnership building – how to communicate the vision and strategic direction for policies, strategies and interventions, and how strategic alliances and partnerships can be built and sustained.  D.2.1. Develop and implement a public health policy/strategy/intervention based on standard public health  methods and guidelines, including e.g.:  D.2.1.7. Communicate effectively and motivate people to engage in change in the organisation and support learning and development of staff.  E.1.6. The basic theories underlying communication skills – the basic principles of:  E.1.6.1. Learning processes;  E.1.6.2. Strategic communication;  E.1.6.3. Marketing;  E.2.2. Communicate effectively public health messages – including risk analysis and priority-setting  issues - to lay, professional, academic and political audiences, by use of modern media, e.g. written media,  audio-visual techniques and internet-based social media tools  E.1.6.4. Target group specific communication.  E.2.8. Write a policy proposal, including:  E.2.8.1. Title page;  E.2.8.2. The concrete health challenge;  E.2.8.3. Scientific background and consequential policy options;  E.2.8.4. Policy recommendations;  E.2.8.5. Communication plan;  E.2.8.6. References. |
| **WHO - ASPHER Competency Framework for the Public Health Workforce in the European Region** | World Health Organization (WHO) and Association of Schools of Public Health in the European Region (ASPHER) | https://apps.who.int/iris/bitstream/handle/10665/347866/WHO-EURO-2020-3997-43756-61569-eng.pdf?sequence=1&isAllowed=y | 7.1 Communicates strategically by defining the target audience, listening and developing audience-appropriate messaging.  7.2 Communicates and shares information and responsibility effectively at different organizational levels to gain political commitment, policy support and social acceptance for a health goal or programme.  7.3 Communicates facts and evidence effectively within the context of translating science and evidence into practice and policy for various actors in the system and populations of concern to increase the effectiveness of responses to risks, threats and damages to health.  7.4 Communicates health messages (including risks to health) effectively (both in writing and verbally) through a range of modern media and social marketing to lay, professional, academic and political audiences.  7.5 Understands and applies cultural awareness and sensitivity in communication with diverse populations.  7.6 Communicates with respect when representing professional opinions and encourages other team members, including community members and patients, to express their opinions and contribute to decision-making.  7.7 Prepares and delivers outputs to facilitate communication within and between organizations such as meeting agendas, presentations, reports and project dissemination.  7.8 Advocates for healthy public policies and services that promote and protect the health and well-being of individuals and communities. |
| **Core Competencies for Public Health Professionals** | The Council on Linkages Between Academia and Public Health Practice | https://www.phf.org/resourcestools/Documents/Core_Competencies_for_Public_Health_Professionals_2021October.pdf | 3.1 Determines communication strategies.  3.2 Communicates with internal and external audiences.  3.2 Communicates with internal and external audiences.  3.3 Responds to information, misinformation, and disinformation.  3.4 Facilitates communication among individuals, groups, and organizations. |
| **Public Health Skills and Knowledge Framework 2016** | Public Health England, Public Health Wales, NHS Scotland, and the Public Health Agency of Nothern Ireland | https://www.gov.uk/government/publications/public-health-skills-and-knowledge-framework-phskf | C2.1 Manage public perception and convey key messages using a range of media processes.  C2.2 Communicate sometimes complex information and concepts (including health outcomes, inequalities and life expectancy) to a diversity of audiences using different methods.  C2.3 Facilitate dialogue with groups and communities to improve health literacy and reduce inequalities using a range of tools and technologies.  C2.4 Apply the principles of social marketing, and/or behavioural science, to reach specific groups and communities with enabling information and ideas.  C2.5 Consult, and listen to individuals, groups and communities likely to be affected by planned intervention or change. |
| **New Zealand College of Public Health Medicine Training Curriculum** | Health Promotion Forum of New Zealand | https://hpfnz.org.nz/assets/Health-Promotion-Competencies-Final.pdf | 4.1 Use culturally appropriate methods and techniques to communicate effectively with specific groups in a range of settings.  4.2 Use interpersonal communication and group work skills to facilitate individuals, groups, communities and organisations to improve health and address health inequities.  4.3 Use concepts of health literacy and effective communication skills, including written, verbal, non-verbal, listening and information technology.  4.4 Use electronic and other new media to receive and disseminate health promotion information. |
| **Health Promotion Competencies for Aotearoa New Zealand** | Health Promotion Forum of New Zealand | https://hpfnz.org.nz/assets/Health-Promotion-Competencies-Final.pdf | 4.1 Use culturally appropriate methods and techniques to communicate effectively with specific groups in a range of settings.  4.2 Use interpersonal communication and group work skills to facilitate individuals, groups, communities and organisations to improve health and address health inequities.  4.3 Use concepts of health literacy and effective communication skills, including written, verbal, non-verbal, listening and information technology.  4.4 Use electronic and other new media to receive and disseminate health promotion information. |
| **Generic Competencies for Public Health in Aotearoa-New Zealand** | Public Health Association of New Zealand | https://www.pha.org.nz/page-18201 | 8.1 Listens actively.  8.2 Uses different communication styles to facilitate understanding accommodate.  8.3 Uses oral communication effectively in a range of contexts.  8.4 Communicates clearly in writing for the given context.  8.5 Consults with others in a range of settings. |
| **Areas of Responsibility, Competencies, and Sub-Competencies for Health Education Specialist Practice Analysis II 2020** | National Commission for Health Education Credentialing | https://www.sophe.org/wp-content/uploads/2020/04/HESPA-II-AREAS-OF-RESPONSIBILITIES.pdf | 6.1 Determine factors that affect communication with the identified audience(s).  6.2 Determine communication objective(s) for audience(s).  6.3 Develop message(s) using communication theories and/or models.  6.4 Select methods and technologies used to deliver message(s).  6.5 Deliver the message(s) effectively using the identified media and strategies.  6.6 Evaluate communication. |
| **IUHPE Core Competencies and Professional Standards for Health Promotion** | Australian Health Promotion Association | http://www.ukphr.org/wp-content/uploads/2017/02/Core_Competencies_Standards_linkE.pdf | 4.1 Use effective communication skills including written, verbal, nonverbal, listening skills and information technology.  4.2 Use electronic and other media to receive and disseminate health promotion information.  4.3 Use culturally appropriate communication methods and techniques for specific groups and settings.  4.4 Use interpersonal communication and groupwork skills to facilitate individuals, groups, communities and organisations to improve health and reduce health inequities. |
| **Core Competencies for Health Promotion Practitioners** | Australian Health Promotion Association | <http://healthpromotionscholarshipswa.org.au/wp-content/uploads/2014/05/core-competencies-for-hp-practitioners.pdf> | 3.1 write reports for a variety of audiences and purposes including papers for peer reviewed journals, in-house reports, program plans and program update reports;  3.2 write for professional audiences;  3.3 write for lay audiences;  3.4 write submissions, grants or applications for funding;  3.5 write for newspapers including media releases;  3.6 apply interpersonal skills (negotiation, team work, motivation, conflict management, decision making, and problem solving skills);  3.7 facilitate meetings;  3.8 debate health-related issues using evidence-based arguments;  3.9 give presentations on health promotion programs or topics at workshops or conferences;  3.10 interpret information for professional, non professional and community audiences; and  3.11 use current technology to communicate effectively. |

*The subfunctions/subcompetencies described in relevant frameworks were included in our analysis but omitted from this table

**Supplementary Table 2** Job/Role of Survey Participants (n=378)

| **Variable/Characteristic** | **Count (n)** | **Frequency (%)**† |
| --- | --- | --- |
|  |  |  |
| **Job Title** | **n** | **%** |
| Public Health Nurse | 72 | 19.0% |
| Manager, Director, Supervisor, or Team Lead | 64 | 16.9% |
| Public Health Promoter | 47 | 12.4% |
| Epidemiologist | 34 | 9.0% |
| Public Health Inspector | 27 | 7.1% |
| Policy Analyst | 22 | 5.8% |
| Program Coordinator | 17 | 4.5% |
| Communication Specialist | 13 | 3.4% |
| Public Health Nutritionist/Dietitian | 9 | 2.4% |
| Health Analyst | 8 | 2.1% |
| Program Evaluator | 5 | 1.3% |
| Research Assistant | 5 | 1.3% |
| Medical Officer of Health/Associate Medical Officer of Health | 4 | 1.1% |
| Practicum Student | 4 | 1.1% |
| Chief Executive Officer or Chief Administrative Officer | 3 | 0.8% |
| Program Analyst | 3 | 0.8% |
| Physician | 2 | 0.5% |
| Research Coordinator | 2 | 0.5% |
| Infection Prevention and Control Practitioner | 1 | 0.3% |
| Other; Please specify: | 35 | 9.3% |
| Choose not to respond | 1 | 0.3% |
|  |  |  |
| **Communication Focused Role** | **n** | **%** |
| No | 265 | 70.1% |
| Yes | 113 | 29.9% |

† Percentages were rounded to one decimal place; category totals may not add to 100%

**Supplementary Table 3** Workforce Agreement with Communication Competency Statements Between Communication Focused Roles (n=113) and Non-Communication Focused Roles (n=265) (See Table 2 for full statements). Bolded statements have group differences for the strongly agree category which are in the top quartile (>|17.0|%)

| **Proposed Statement** | **Total Disagreement** | | | **Agree** | | | **Strongly Agree** | | |
| --- | --- | --- | --- | --- | --- | --- | --- | --- | --- |
|  | **Comm** | **Non-Comm** | **∆** | **Comm** | **Non-Comm** | **∆** | **Comm** | **Non-Comm** | **∆** |
| C1 | 5.3% | 9.1% | -3.7% | 39.8% | 46.0% | -6.2% | 53.1% | 43.4% | 9.7% |
| C2 | 5.3% | 6.0% | -0.7% | 34.5% | 37.7% | -3.2% | 57.5% | 54.0% | 3.5% |
| C3 | 4.4% | 5.3% | -0.9% | 35.4% | 35.5% | -0.1% | 58.4% | 57.0% | 1.4% |
| C4 | 5.3% | 6.0% | -0.7% | 38.1% | 46.0% | -7.9% | 55.8% | 46.0% | 9.8% |
| **C5** | 4.4% | 5.7% | -1.2% | 25.7% | 42.6% | -16.9% | 69.0% | 49.8% | **19.2%** |
| C6 | 6.2% | 8.3% | -2.1% | 35.4% | 37.0% | -1.6% | 57.5% | 53.2% | 4.3% |
| C7 | 1.8% | 2.6% | -0.9% | 22.1% | 34.3% | -12.2% | 76.1% | 60.8% | 15.3% |
| **C8** | 6.2% | 9.1% | -2.9% | 31.0% | 44.9% | -13.9% | 61.9% | 43.8% | **18.1%** |
| C9 | 2.7% | 2.6% | 0.0% | 15.9% | 27.2% | -11.3% | 80.5% | 68.3% | 12.2% |
| C10 | 5.3% | 6.0% | -0.7% | 37.2% | 36.2% | 1.0% | 56.6% | 55.5% | 1.1% |
| C11 | 7.1% | 11.3% | -4.2% | 46.0% | 50.2% | -4.2% | 46.0% | 36.6% | 9.4% |
| C12 | 1.8% | 2.3% | -0.5% | 29.2% | 34.0% | -4.8% | 68.1% | 61.5% | 6.6% |
| **C13** | 6.2% | 14.7% | -8.5% | 44.2% | 52.5% | -8.3% | 48.7% | 30.6% | **18.1%** |
| **C14** | 4.4% | 2.6% | 1.8% | 29.2% | 47.2% | -18.0% | 65.5% | 47.9% | **17.6%** |
| C15 | 2.7% | 4.2% | -1.5% | 32.7% | 41.1% | -8.4% | 62.8% | 52.5% | 10.3% |
| C16 | 1.8% | 3.4% | -1.6% | 23.0% | 25.7% | -2.7% | 74.3% | 68.7% | 5.6% |
| C17 | 3.5% | 6.8% | -3.3% | 23.9% | 36.2% | -12.3% | 69.9% | 54.3% | 15.6% |
| C18 | 7.1% | 8.7% | -1.6% | 36.3% | 41.9% | -5.6% | 54.9% | 47.5% | 7.4% |
| C19 | 2.7% | 5.7% | -3.0% | 26.5% | 35.8% | -9.3% | 69.0% | 56.6% | 12.4% |
| **C20** | 6.2% | 9.4% | -3.2% | 29.2% | 43.8% | -14.6% | 61.9% | 44.5% | **17.4%** |
| **C21** | 6.2% | 3.4% | 2.8% | 19.5% | 39.2% | -19.7% | 72.6% | 55.5% | **17.1%** |

† Percentages were rounded to one decimal place and non-responses were omitted; category totals may not add to 100%

**Supplementary Table 4** Workforce agreement with communication competency statements between professionals who have been in their role for 5 years or less (n=218) compared to 6 years or more (n=160) (See Table 2 for full statements). Bolded statements have group differences for the strongly agree category which are in the top quartile (>|4.7|%)

| **Proposed Statement** | **Total Disagreement** | | | **Agree** | | | **Strongly Agree** | | |
| --- | --- | --- | --- | --- | --- | --- | --- | --- | --- |
|  | **6 or More**  **Years** | **5 or Less**  **Years** | **∆** | **6 or More**  **Years** | **5 or Less**  **Years** | **∆** | **6 or More**  **Years** | **5 or Less**  **Years** | **∆** |
| C1 | 10.0% | 6.4% | 3.6% | 45.0% | 43.6% | 1.4% | 45.0% | 47.2% | -2.2% |
| C2 | 8.1% | 4.1% | 4.0% | 38.1% | 35.8% | 2.3% | 52.5% | 56.9% | -4.4% |
| C3 | 5.6% | 4.6% | 1.0% | 35.6% | 35.3% | 0.3% | 58.1% | 56.9% | 1.2% |
| C4 | 6.9% | 5.0% | 1.8% | 46.9% | 41.3% | 5.6% | 46.3% | 50.9% | -4.7% |
| C5 | 3.1% | 6.9% | -3.8% | 42.5% | 33.9% | 8.6% | 53.8% | 56.9% | -3.1% |
| C6 | 7.5% | 7.8% | -0.3% | 40.0% | 33.9% | 6.1% | 52.5% | 56.0% | -3.5% |
| **C7** | 1.3% | 3.2% | -2.0% | 30.0% | 31.2% | -1.2% | 68.1% | 63.3% | **4.8%** |
| C8 | 10.6% | 6.4% | 4.2% | 40.0% | 41.3% | -1.3% | 48.8% | 49.5% | -0.8% |
| C9 | 2.5% | 2.8% | -0.3% | 23.8% | 23.9% | -0.1% | 73.1% | 71.1% | 2.0% |
| C10 | 4.4% | 6.9% | -2.5% | 36.9% | 36.2% | 0.6% | 58.1% | 54.1% | 4.0% |
| **C11** | 11.3% | 9.2% | 2.1% | 52.5% | 46.3% | 6.2% | 35.6% | 42.2% | **-6.6%** |
| C12 | 2.5% | 1.8% | 0.7% | 35.6% | 30.3% | 5.3% | 61.3% | 65.1% | -3.9% |
| C13 | 11.9% | 12.4% | -0.5% | 51.9% | 48.6% | 3.3% | 35.6% | 36.2% | -0.6% |
| **C14** | 3.1% | 3.2% | -0.1% | 45.6% | 39.0% | 6.6% | 50.0% | 55.5% | **-5.5%** |
| C15 | 1.9% | 5.0% | -3.2% | 40.6% | 37.2% | 3.5% | 56.9% | 54.6% | 2.3% |
| C16 | 1.9% | 3.7% | -1.8% | 28.1% | 22.5% | 5.6% | 69.4% | 71.1% | -1.7% |
| C17 | 6.9% | 5.0% | 1.8% | 33.8% | 31.7% | 2.1% | 58.1% | 59.6% | -1.5% |
| **C18** | 6.3% | 9.6% | -3.4% | 36.9% | 42.7% | -5.8% | 56.9% | 44.5% | **12.4%** |
| C19 | 5.6% | 4.1% | 1.5% | 33.1% | 33.0% | 0.1% | 61.3% | 59.6% | 1.6% |
| C20 | 8.1% | 8.7% | -0.6% | 42.5% | 37.2% | 5.3% | 48.1% | 50.9% | -2.8% |
| **C21** | 5.6% | 3.2% | 2.4% | 36.3% | 31.2% | 5.1% | 57.5% | 62.8% | **-5.3%** |

† Percentages were rounded to one decimal place and non-responses were omitted; category totals may not add to 100%

**Supplementary Table 5** Communication and Health Communication Training in the Public Health Workforce

| **Existing Training** | **n (%)** |
| --- | --- |
|  |  |
| Communication Training Received* |  |
| Informal | 238 (63.0%) |
| Formal | 152 (40.2%) |
| None | 103 (27.2%) |
| No Response | 8 (2.1%) |
|  |  |
| Health Communication Training Received* |  |
| Informal | 207 (54.8%) |
| Formal | 141 (37.3%) |
| None | 107 (28.3%) |
| No Response | 8 (2.1%) |
|  |  |
| Current Health Communication Training Opportunities |  |
| Inadequate | 324 (85.7%) |
| Adequate | 46 (12.2%) |
| No Response | 8 (2.1%) |
| **Interest in Training Opportunities** |  |
|  |  |
| Interest in Resources/Future Training* |  |
| Webinar | 317 (83.9%) |
| Digital Content Hub (Website) | 238 (63.0%) |
| Digital Resource (PDF) | 235 (62.2%) |
| Textbook (e-book or print) | 78 (20.6%) |
| Certificate in Health Communication (online) | 307 (81.2%) |
| Graduate Diploma in Health Communication (online) | 146 (38.6%) |
| Graduate Diploma in Health Communication (in-person) | 54 (14.3%) |
| Graduate Degree (Master of Public Health) with Health Communication Specialization (in person) | 75 (19.8%) |
| Graduate Degree (MSc) in Health Communication (in person and online) | 81 (21.4%) |
| Graduate Degree (PhD) in Health Communication (in person and online) | 49 (13.0%) |
| No Response | 15 (4.0%) |

* Multiple categories were possible; category totals may exceed 100%.

† Percentages were rounded to one decimal place; category totals may exceed 100%
