# Supplementary material for: Modernizing public health communication competencies in Canada: A survey of the Canadian public health workforce
Source: Can J Public Health. 2024 May 16;115(4):664–79. doi: 10.17269/s41997-024-00890-w (PMC11303361; doi:10.17269/s41997-024-00890-w)
Supplement: Supplementary file 2 — Supplementary file2 (DOCX 41 KB) [file 41997_2024_890_MOESM2_ESM.docx]

**English Questionnaire:**

What is your job title? (Please select the most appropriate option)

- Chief Executive Officer or Chief Administrative Officer
- Medical Officer of Health/Associate Medical Officer of Health
- Manager, Director, or Team Lead
- Communication Specialist
- Emergency Planner
- Epidemiologist
- Health Analyst
- Infection Prevention and Control Practitioner
- Physician
- Policy Analyst
- Practicum Student
- Program Analyst
- Program Coordinator
- Program Evaluator
- Public Health Dentist
- Public Health Inspector
- Public Health Nurse
- Public Health Nutritionist/Dietitian
- Public Health Promoter
- Research Assistant
- Research Coordinator
- Veterinarian
- Other; Please specify: __________________________________________________
- Choose not to respond

How long have you been in your current role?

- Less than 1 year
- 1-5 years
- 6-10 years
- More than 10 years

Are you currently in a communication-focused role (e.g., Communications Specialist, Manager of Communications, Health Communication/Promotion)?

- Yes
- No

What type of organization do you work for?

- Municipal government
- Local Public Health unit
- Community Health Centre
- Provincial government/agency
- Federal government/agency
- Private industry
- Academia
- Non-governmental organization
- Other; Please specify: __________________________________________________
- Choose not to respond

What Province(s)/Territory(ies) is your organization located in? Please select all that apply.

- National
- Alberta
- British Columbia
- Manitoba
- New Brunswick
- Newfoundland and Labrador
- Northwest Territories
- Nova Scotia
- Nunavut
- Ontario
- Prince Edward Island
- Quebec
- Saskatchewan
- Yukon
- Choose not to respond

Does your organization identify Indigenous peoples and/or Indigenous communities as priority populations?

- Yes
- No

How would you describe your gender identity?

- Agender
- Gender fluid
- Gender queer
- Man (includes cis-men, trans-men, and everyone else who identifies as a man)
- Non-binary
- Woman (includes cis-women, trans-women, and everyone else who identifies as a woman)
- Prefer to self-describe: __________________________________________________
- Choose not to respond

Which of the following best describes your HIGHEST level of education? (If you hold both a graduate degree and a professional degree, please select the highest education most relevant to your current role)

- High school education
- Some college/university
- Completed college/university
- Some graduate education
- Completed graduate education
- Professional degrees

Have you received training in **communication** (i.e., general communication, marketing, public relations, media training)? (Select all that apply.)

- Yes, formal training in communication (e.g., education, certificates, courses); Please describe: __________________________________________________
- Yes, informal training in communication (e.g., on-the-job training, attending webinars, seeking resources, self-taught); Please describe: __________________________________________________
- No

Have you received training in **health communication** (i.e., communication about health with various audiences)? Please select all that apply.

- Yes, formal training in health communication (e.g., education, certificates, courses); Please describe: __________________________________________________
- Yes, informal training in health communication (e.g., on-the-job training, attending webinars, seeking resources, self-taught); Please describe: __________________________________________________
- No

How important do you think **communication** is as a competency for **Public Health professionals**?

- Not Important (1)
- Somewhat Important (2)
- Important (3)
- Very Important (4)

Has the COVID-19 pandemic influenced how important you think **communication** is as a competency for **Public Health professionals**?

- Much less important (1)
- Somewhat less important (2)
- About the same importance (3)
- Somewhat more important (4)
- Much more important (5)

How would you rate **your overall communication competence**?

- Not Competent (1)
- Somewhat Competent (2)
- Competent (3)
- Very Competent (4)

How would you rate **your competence** (skills, knowledge, attitudes/values, and behaviours) in the following PHAC communication competencies?

| Communicate effectively with individuals, families, groups, communities, and colleagues. | Not Competent (1) | Somewhat Competent (2) | Competent (3) | Very Competent (4) |
| --- | --- | --- | --- | --- |
| Interpret information for professional, non-professional and community audiences. | Not Competent (1) | Somewhat Competent (2) | Competent (3) | Very Competent (4) |
| Mobilize individuals and communities by using appropriate media, community resources and social marketing techniques. | Not Competent (1) | Somewhat Competent (2) | Competent (3) | Very Competent (4) |
| Use current technology to communicate effectively. | Not Competent (1) | Somewhat Competent (2) | Competent (3) | Very Competent (4) |

How competent are you at each of the following **types of communication**?

| Written Communication (e.g., documents, pamphlets, articles, emails, instant messages) | Not Competent (1) | Somewhat Competent (2) | Competent (3) | Very Competent (4) |
| --- | --- | --- | --- | --- |
| Verbal/Oral Communication (e.g., oral presentations, phone calls, virtual or in-person conversations) | Not Competent (1) | Somewhat Competent (2) | Competent (3) | Very Competent (4) |
| Visual Communication (e.g., info-graphics, videos, photographs, diagrams, data visualization) | Not Competent (1) | Somewhat Competent (2) | Competent (3) | Very Competent (4) |

How competent are you at communicating with each of the following **audiences**?

| Professional Public Health Audiences (e.g., internal audiences, Public Health peers/colleagues, Public Health researchers) | Not Competent (1) | Somewhat Competent (2) | Competent (3) | Very Competent (4) |
| --- | --- | --- | --- | --- |
| Professional Non-Public Health Audiences (e.g., news, media, politicians) | Not Competent (1) | Somewhat Competent (2) | Competent (3) | Very Competent (4) |
| Public Audiences (e.g., individuals/patients, families, groups, communities, populations) | Not Competent (1) | Somewhat Competent (2) | Competent (3) | Very Competent (4) |
| Partner Audiences (e.g., non-Public Health partners) | Not Competent (1) | Somewhat Competent (2) | Competent (3) | Very Competent (4) |
| Other; Please specify: | Not Competent (1) | Somewhat Competent (2) | Competent (3) | Very Competent (4) |

How competent are you at using the following **channels** to communicate?

| Social Media | Not Competent (1) | Somewhat Competent (2) | Competent (3) | Very Competent (4) |
| --- | --- | --- | --- | --- |
| Website/Blog Maintenance/Creation | Not Competent (1) | Somewhat Competent (2) | Competent (3) | Very Competent (4) |
| Online Resources/Websites | Not Competent (1) | Somewhat Competent (2) | Competent (3) | Very Competent (4) |
| Software/Apps | Not Competent (1) | Somewhat Competent (2) | Competent (3) | Very Competent (4) |
| Webinars | Not Competent (1) | Somewhat Competent (2) | Competent (3) | Very Competent (4) |
| Conferences | Not Competent (1) | Somewhat Competent (2) | Competent (3) | Very Competent (4) |
| Presentations | Not Competent (1) | Somewhat Competent (2) | Competent (3) | Very Competent (4) |
| News Media | Not Competent (1) | Somewhat Competent (2) | Competent (3) | Very Competent (4) |
| Community Networks | Not Competent (1) | Somewhat Competent (2) | Competent (3) | Very Competent (4) |
| Reports/Documents | Not Competent (1) | Somewhat Competent (2) | Competent (3) | Very Competent (4) |
| Emails | Not Competent (1) | Somewhat Competent (2) | Competent (3) | Very Competent (4) |
| Phone Calls | Not Competent (1) | Somewhat Competent (2) | Competent (3) | Very Competent (4) |
| Virtual Meetings | Not Competent (1) | Somewhat Competent (2) | Competent (3) | Very Competent (4) |
| In-Person | Not Competent (1) | Somewhat Competent (2) | Competent (3) | Very Competent (4) |
| Other; Please specify: | Not Competent (1) | Somewhat Competent (2) | Competent (3) | Very Competent (4) |

The following list contains 21 modernized competency statements for communication in public health. Please review the list and **consider how much you agree with each statement as a communication competency** for the Canadian public health workforce. Please consider using the full range of response options as you feel appropriate. Please rank each statement from 1 (Strongly Disagree) - 4 (Strongly Agree):

| Apply key relevant interdisciplinary **theories, frameworks, toolkits**, and **best practice guidelines** to develop health communication initiatives. | 1 | 2 | 3 | 4 |
| --- | --- | --- | --- | --- |
| Ground health communication efforts **in appropriate philosophical** and **critical perspectives**, including **social and health justice, health equity,** and public health **ethics**. | 1 | 2 | 3 | 4 |
| Foster attitudes and approaches for fair, equitable, and inclusive communication (e.g., **empathy, respect, solidarity, compassion, reciprocity,** and **reflexivity**). | 1 | 2 | 3 | 4 |
| Use an **audience-centered, participatory** approach to develop communication initiatives (e.g., **co-design, community-based approaches,** and **two-way communication**). | 1 | 2 | 3 | 4 |
| Identify and integrate factors that influence how different audiences **find, understand, use, apply,** and **act** on health information . | 1 | 2 | 3 | 4 |
| Apply the necessary **knowledge synthesis** and **research skills** to develop evidence-informed, theory-based health communication initiatives. | 1 | 2 | 3 | 4 |
| Select and apply appropriate approaches and tools for the design of health messages to ensure they are **accurate, clear, accessible, credible,** and **understandable**. | 1 | 2 | 3 | 4 |
| Design health communication initiatives that **inform** and/or **persuade** to impact knowledge, attitudes, intentions, behaviours, self-efficacy, resilience and individual and population health. | 1 | 2 | 3 | 4 |
| Design health messaging that is **transparent, trustworthy, tailored,** and, where applicable, delivered by an appropriate **messenger** or source . | 1 | 2 | 3 | 4 |
| Identify and use current and emerging **communication channels, settings,** and **technologies** to meet audience information needs. | 1 | 2 | 3 | 4 |
| Monitor **information ecosystems** to identify and address related challenges (e.g., information seeking behaviours, information availability and accessibility, and mis/disinformation ). | 1 | 2 | 3 | 4 |
| Plan evidence-based communication initiatives with **clear goals** and **objectives**, appropriate **timing, partnerships**, and **resources**, that **meet community needs** . | 1 | 2 | 3 | 4 |
| Identify audiences and generate **nuanced audience profiles** (e.g., type, level, segmentation) to guide communication. | 1 | 2 | 3 | 4 |
| Implement the appropriate approaches for communication initiatives based on **context** and **audience characteristics**. | 1 | 2 | 3 | 4 |
| **Plan** and **implement** appropriate **evaluation** of communication initiatives . | 1 | 2 | 3 | 4 |
| Communicate in a **culturally competent** and safe manner in a way that is informed by the relationship between culture, language, and health . | 1 | 2 | 3 | 4 |
| Attend carefully to the **barriers** to and the **unintended consequences** of impactful health communication, which may limit effectiveness and exacerbate harms and health disparities. | 1 | 2 | 3 | 4 |
| Effectively apply communication skills in **activism, advocacy,** and **partnerships** to improve individual health, population health, and health equity . | 1 | 2 | 3 | 4 |
| Mobilize **evidence** and **different ways of knowing** to **inform policy** and **practice** decisions in public health. | 1 | 2 | 3 | 4 |
| Use **specialized communication** (e.g., crisis, risk, clinical) to interpret, translate, and tailor **complex information** to **guide action**. | 1 | 2 | 3 | 4 |
| Create and use a **range of communication materials** (e.g., policy brief, news media article, presentations, recordings, stories) and **types** (e.g., oral, written, visual) that meet audience needs and reflect their values. | 1 | 2 | 3 | 4 |

Are there any other competencies (skills, knowledge, attitudes/values, or behaviours) that you would like to see included in a list of Communication Competencies for Public Health?

________________________________________________________________

Is there anything else you would like to add or share regarding Communication Competencies for Public Health?

________________________________________________________________

In your opinion, are there adequate Canadian Public Health training opportunities for health communication?

- Yes
- No

Would you be interested in any specialized resources or training opportunities in Health Communication to enhance communication competence? Please select all that apply.

- Webinar(s)
- Digital content hub (website)
- Digital resource (PDF guidebook)
- Textbook (e-book or print)
- Certificate in Health Communication (online)
- Graduate Diploma in Health Communication (online)
- Graduate Diploma in Health Communication (in-person)
- Graduate Degree (Master of Public Health) with Health Communication Specialization (in person)
- Graduate Degree (MSc) in Health Communication (in person and online)
- Graduate Degree (PhD) in Health Communication (in person and online)
